# A Novel In Vitro Assay Using Human iPSC-Derived Sensory Neurons to Evaluate the Effects of External Chemicals on Neuronal Morphology: Possible Implications in the Prediction of Abnormal Skin Sensation

**DOI:** 10.3390/ijms221910525

**Published:** 2021-09-29

**Authors:** Masahiko Satoh, Tamie Suzuki, Tetsuhito Sakurai, Sumika Toyama, Yayoi Kamata, Shinya Kondo, Yasushi Suga, Mitsutoshi Tominaga, Kenji Takamori

**Affiliations:** 1 FANCL Research Institute, FANCL Corporation, 12–13 Kamishinano Totsuka, Yokohama 244-0806, Kanagawa, Japan; sato_masahiko@fancl.co.jp (M.S.); tamisuzuki@fancl.co.jp (T.S.); tesakurai@fancl.co.jp (T.S.); kondo_shinya@fancl.co.jp (S.K.); 2 Anti-Aging Skin Research Laboratory, Juntendo University Graduate School of Medicine, 2-1-1 Tomioka, Urayasu 279-0021, Chiba, Japan; ykamata@juntendo.ac.jp (Y.K.); ysuga@juntendo.ac.jp (Y.S.); tominaga@juntendo.ac.jp (M.T.); 3 Juntendo Itch Research Center (JIRC), Institute for Environmental and Gender-Specific Medicine, Juntendo University Graduate School of Medicine, 2-1-1 Tomioka, Urayasu 279-0021, Chiba, Japan; su-toyama@juntendo.ac.jp; 4 Department of Dermatology, Juntendo Urayasu Hospital, 2-1-1 Tomioka, Urayasu 279-0021, Chiba, Japan

**Keywords:** safety testing, sensitive skin, sensory neuron, preservatives

## Abstract

Neuronal morphological changes in the epidermis are considered to be one of causes of abnormal skin sensations in dry skin-based skin diseases. The present study aimed to develop an in vitro model optimised for human skin to test the external factors that lead to its exacerbation. Human-induced pluripotent stem cell-derived sensory neurons (hiPSC-SNs) were used as a model of human sensory neurons. The effects of chemical substances on these neurons were evaluated by observing the elongation of nerve fibers, incidence of blebs (bead-like swellings), and the expression of nicotinamide mononucleotide adenylyl transferase 2 (NMNAT2). The nerve fiber length increased upon exposure to two common cosmetic preservatives—methylparaben and phenoxyethanol—but not to benzo[a]pyrene, an air pollutant at the estimated concentrations in the epidermis. Furthermore, the incidence of blebs increased upon exposure to benzo[a]pyrene. However, there was a decrease in the expression of NMNAT2 in nerve fibers, suggesting degenerative changes. No such degeneration was found after methylparaben or phenoxyethanol at the estimated concentrations in the epidermis. These findings suggest that methylparaben and phenoxyethanol promote nerve elongation in hiPSC-SNs, whereas benzo[a]pyrene induces nerve degeneration. Such alterations may be at least partly involved in the onset and progression of sensitive skin.

## 1. Introduction

Sensitive skin is defined as a syndrome involving the onset of unpleasant sensations, such as burning, stinging, tingling, pricking, or itching, in response to stimuli that do not normally result in such sensations [1]. The number of people who consider themselves to have sensitive skin is increasing [2,3], and the causes for this increase include changes in the environment, eating habits, and increased stress. Sensitive skin is characterised by alterations of neuronal morphology [4,5], and the sensation of itching is the main complaint that people with sensitive skin have [6].

Clinically, atopic dermatitis is dry-skin based skin disease characterized by intense pruritus [7]. The itching leads to scratching, which reduces the barrier function of the skin, resulting in a vicious cycle. Such abnormal skin sensations are also caused in part by neuronal morphological changes, such as epidermal hyperinnervation [8] and neurodegeneration [9]. Recently, sensitization of sensory neurons has been documented to be regulated by certain inflammatory mediators or kinases as well as the association of transient receptor potential vanilloid 1 (TRPV1) and transient receptor potential ankyrin 1 (TRPA1) [10]. However, the exacerbating factors for abnormal skin sensations remain unknown. External chemical substances that can cause stress to the skin include preservatives in cosmetic products and environmental pollutants in the atmosphere. The nerve fibers of the epidermis can be affected by external chemical substances by penetrating the epidermis through the stratum corneum.

The rodent dorsal root ganglion neuron model has been widely used to evaluate how nerve fibers in the skin are affected by their surroundings in vitro. However, there are interspecies differences in responsiveness to chemical substances [11]. Although it is preferable to use human-derived sensory neurons when establishing an in vitro sensory neuron model optimised for human skin, it is ethically difficult to obtain such neurons for experimental use. Therefore, in this study, we used human-induced pluripotent stem cell-derived sensory neurons (hiPSC-SNs) to evaluate the effects of chemical substances and the microenvironment on neuronal morphology in human skin. Herein, we describe that hiPSC-SNs serve as a useful tool for evaluating the ability of cosmetic ingredients to exacerbate abnormal skin sensations.

## 2. Results

### 2.1. Immunocytochemical Characterisation of Differentiated hiPSC-SNs

**Figure 1:**
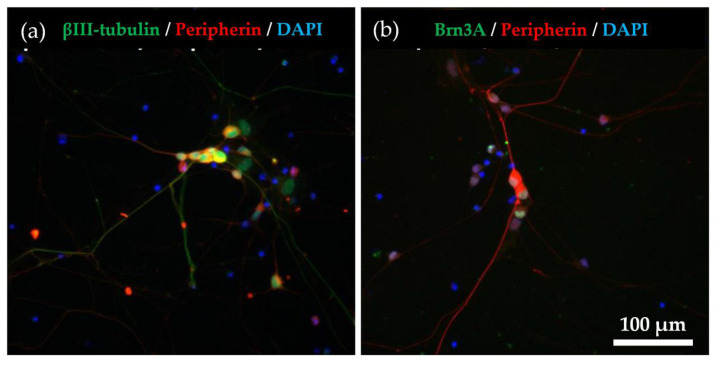

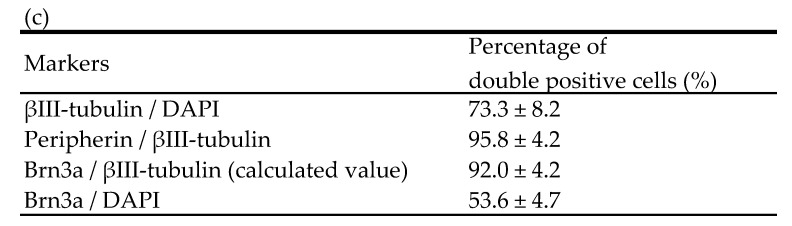
Immunocytochemical characterization for hiPSC-SNs. (**a**) After the culture of hiPSC-SNs for 6 days, neurons expressed βIII-tubulin (green) and peripherin (red). Nuclei were visualised with DAPI (blue). (**b**) The cells expressed peripherin (red) and Brn3a (green). (**c**) Percentages of the βIII-tubulin, peripherin, and DAPI-positive cells. Data are expressed as mean ± standard deviation. hiPSC-SNs: human-induced pluripotent stem cell-derived sensory neurons.

We characterised hiPSC-SNs using combinations of established molecular markers to determine whether the cells differentiated sufficiently into C-fiber neurons. The expression levels of βIII-tubulin (a pan-neuronal marker), Brn3a (a sensory neuronal marker), and peripherin (a C-fiber marker) are commonly used to identify sensory neurons [12,13,14,15]. Using immunocytochemistry, we observed many β-III-tubulin- and peripherin-positive cells (Figure 1a), as well as Brn3a- and peripherin-positive cells (Figure 1b). By manual counting, we estimated Brn3a- and peripherin-positive cells to account for 92.0% and 95.8% of βIII-tubulin-positive cells, respectively (Figure 1c).

### 2.2. Responsiveness of hiPSC-SNs to NGF

**Figure 2:**
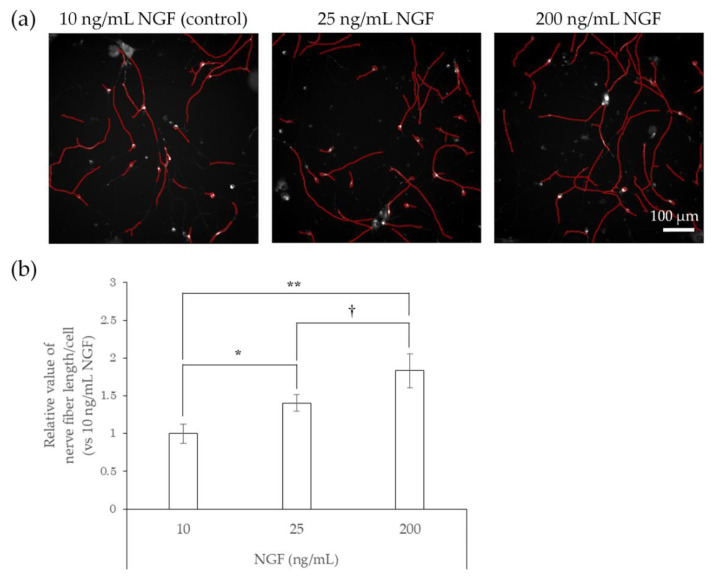
Effects of NGF on nerve fiber elongation of hiPSC-SNs. After culturing for 3 days, hiPSC-SNs were incubated with medium containing NGF for 24 h and stained with NeuO, a neuron-selective probe. (**a**) Representative images with nerve fibers outlined in red were obtained using IN Cell Analyzer 2200. The red lines indicate NeuO-stained nerve fibers. (**b**) Nerve fiber length per cell versus 10 ng/mL NGF after incubation with various doses of NGF. Each value represents the mean of three to four wells. * *p* < 0.05, ** *p* < 0.001, vs. 10 ng/mL NGF (Tukey’s test); † *p* < 0.05, vs. 25 ng/mL NGF (Tukey’s test). NGF: nerve growth factor.

Epidermal hyperinnervation is thought to be caused by nerve elongation factors, such as NGF [16]. To assess the value of hiPSC-SNs as an in vitro model for testing the effects of environmental factors on hyperinnervation, we examined whether the cells responded to NGF. In the test, hiPSC-SNs were cultured with medium containing NGF for 24 h and stained with NeuO, a neuron-selective probe [17], and the length of nerve fibers was then quantified using the IN Cell Analyzer 2200 system and the IN Cell Investigator 1.6. The nerve fibers of hiPSC-SNs were elongated in the presence of NGF (Figure 2a), and the relative value of nerve fiber length per cell significantly increased in a dose-dependent manner (Figure 2b).

### 2.3. Elongation of Nerve Fibers in Response to Interaction with Preservatives and Air Pollutant

**Figure 3:**
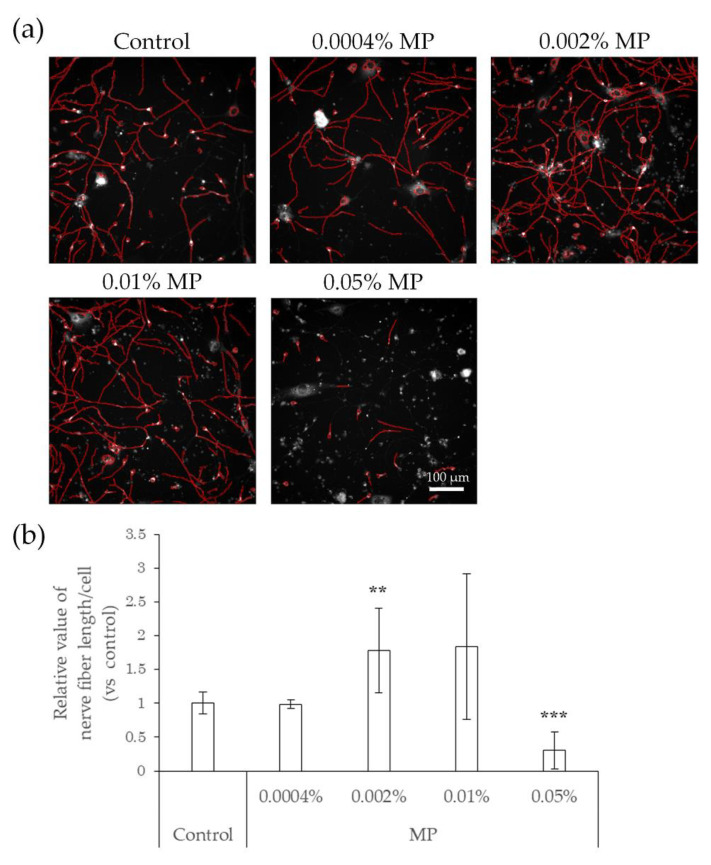
Effects of direct action of MP on nerve fiber elongation of hiPSC-SNs. After culturing for 3 days, hiPSC-SNs were incubated with medium containing MP for 24 h and stained with NeuO. (**a**) Representative images with nerve fibers outlined in red were obtained using IN Cell Analyzer 2200. The red lines indicate NeuO-stained nerve fibers. (**b**) Nerve fiber length per cell versus the control after incubation with various doses of MP. Each value represents the mean ± standard deviation of 4–25 wells. ** *p* < 0.005, *** *p* < 0.001, vs. the control (Steel’s test). MP: methylparaben.

**Figure 4:**
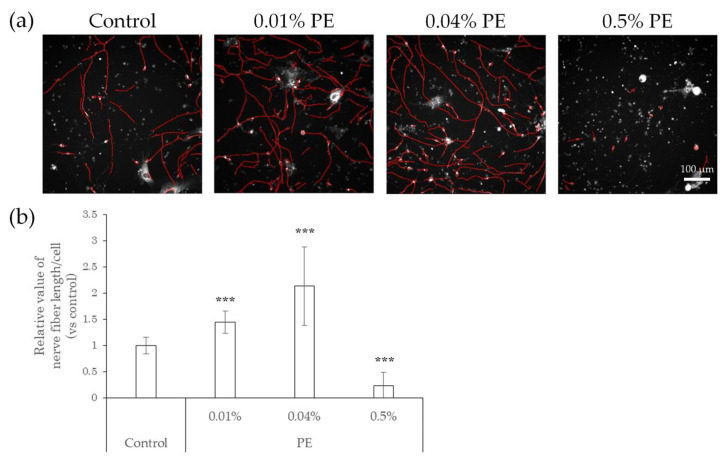
Effects of direct action of PE on nerve fiber elongation of hiPSC-SNs. After culturing for 3 days, hiPSC-SNs were incubated with medium containing methylparaben for 24 h and stained with NeuO. (**a**) Representative images with nerve fibers outlined in red were obtained using IN Cell Analyzer 2200. The red lines indicate NeuO-stained nerve fibers. (**b**) Nerve fiber length per cell versus the control after incubation with various doses of PE. Each value represents the mean ± standard deviation of 7–25 wells. *** *p* < 0.001, vs. the control (Steel’s test). PE: phenoxyethanol.

We next tested whether preservatives, such as methylparaben and phenoxyethanol, or air pollutants, such as benzo[a]pyrene (BaP), affect the elongation of nerve fibers using hiPSC-SNs. In this test, the cells were cultured in medium containing methylparaben, phenoxyethanol, or BaP for 24 h and stained with NeuO, after which their nerve fiber lengths were quantified. In comparison with controls, methylparaben-treated hiPSC-SNs showed no change in nerve fiber length per cell at a concentration of 0.0004%, increased at concentrations of 0.002% and 0.01% (the estimated concentrations reached in the epidermis after exposure to this topical cosmetic agent in previous research [18]), and then decreased at 0.05% (Figure 3). Upon exposure to phenoxyethanol, the nerve fiber length per cell increased at concentrations of 0.01% and 0.04% (the estimated concentrations in the epidermis; in-house standard, data not published), and decreased by 0.5% (Figure 4).

**Figure 5:**
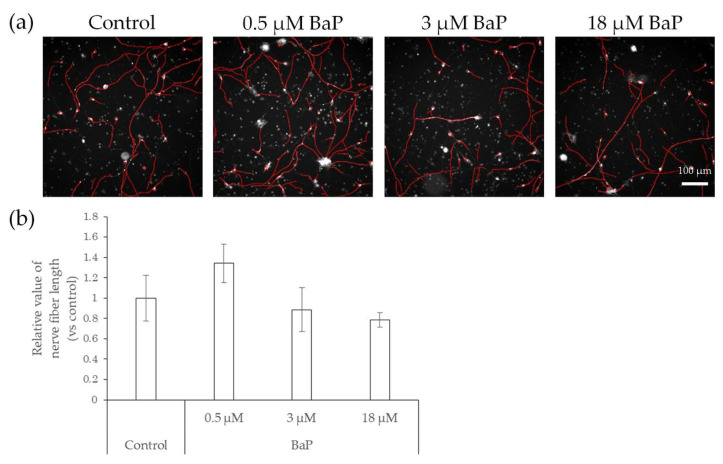
Effects of direct action of BaP on nerve fiber elongation of hiPSC-SNs. After culturing for 3 days, hiPSC-SNs were incubated with a medium containing BaP for 24 h and stained with NeuO. (**a**) Representative images with nerve fibers, outlined in red, were obtained using IN Cell Analyzer 2200. The red lines indicate NeuO-stained nerve fibers. (**b**) Total nerve fiber length versus control after incubation with various doses of BaP. Each value represents the mean ± standard deviation of three to four wells. BaP: benzo[a]pyrene.

Compared with control neurons, neurons exposed to BaP showed a slight but not significant increase in nerve fiber length when exposed to 0.5 μM BaP, which is the probable concentration in the skin, as indicated in previous studies [19,20], and a slight but not significant decrease at concentrations of 3 μM and 18 μM (Figure 5).

### 2.4. Neurodegeneration Due to Interaction with Substances

**Figure 6:**
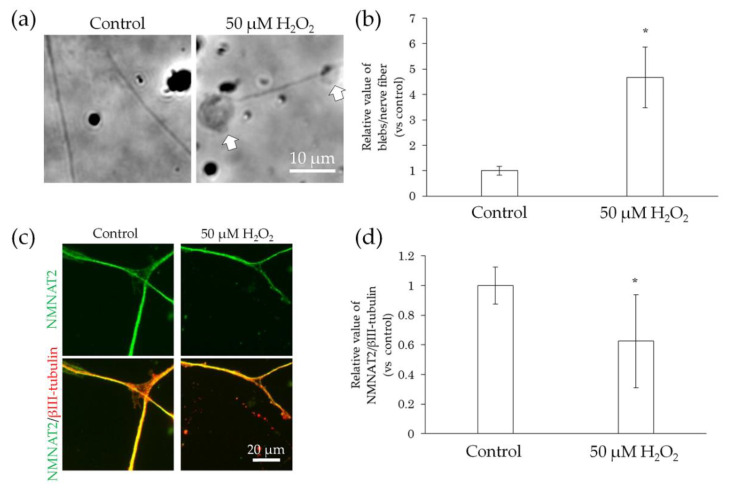
Effect of H_2_O_2_ treatment on bleb formation and NMNAT2 expression in nerve fibers. After culturing for 3 days, the effects of the direct interaction between hiPSC-SN and H_2_O_2_ were assessed. (**a**) Morphological changes in nerve fibers 24 h after treatment with H_2_O_2_. The arrows indicate blebs. (**b**) Number of blebs in 50 μM H_2_O_2_. (**c**) Double labelling of NMNAT2 (red) and βIII-tubulin (green) in nerve fibers with 50 μM H_2_O_2_. (**d**) NMNAT2-immunoreactive (ir) nerve fiber length normalised to βIII-tubulin-ir nerve fiber length in an identical area with 50 μM H_2_O_2_. * *p* < 0.05, vs. absence of H_2_O_2_ (Welch’s t-test). Each value represents the mean ± standard deviation of more than three wells. H_2_O_2_: hydrogen peroxide; NMNAT2: nicotinamide mononucleotide adenylyltransferase 2.

We further examined whether the direct interaction between nerve fibers and preservative or air pollutants caused neurodegeneration. Neurodegeneration affects sensory transmission and can cause abnormal skin sensations, including pain, burning, itching, stinging, and dryness [9,21,22]. Degenerating nerve fibers show an increase in blebs [23,24] and decreased expression of NMNAT2, which is a protein involved in anterograde transport, in the distal stumps of injured neurons before the formation of blebs [23,25]. Therefore, we used the incidence of blebs and NMNAT2 expression on nerve fibers as indicators of neurodegeneration. Given that H_2_O_2_ has been reported to induce degeneration of axons and dendrites in primary cultures of granule cells in the mouse cerebellum [26], we treated hiPSC-SNs with H_2_O_2_. The H_2_O_2_-treated neurons showed increased blebs (Figure 6a,b) and decreased NMNAT2 expression (Figure 6c,d) when compared to the controls.

**Figure 7:**
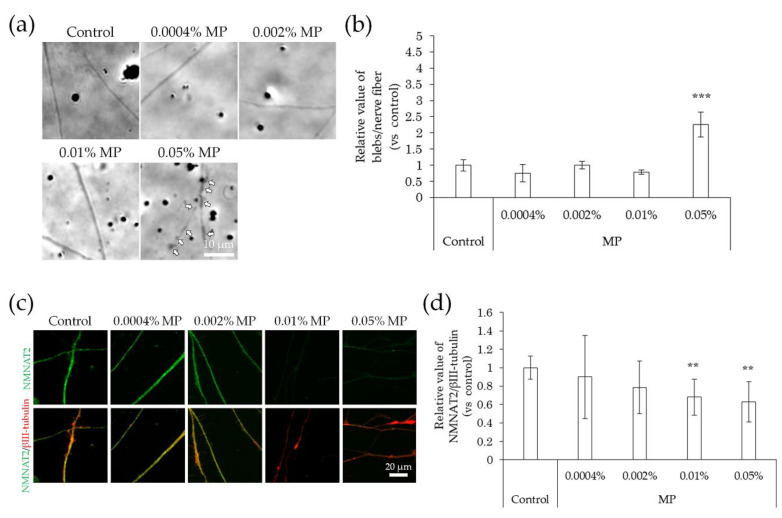
Effects of MP treatment on bleb formation and NMNAT2 expression in nerve fibers. After culturing for 3 days, the effects of the direct interaction between hiPSC-SN and MP were assessed. (**a**) Morphological changes in nerve fibers 24 h after treatment with MP. The arrows indicate blebs. (**b**) The number of blebs at various concentrations of MP. *** *p* < 0.001 vs. absence of MP (Dunnett’s test). (**c**) Double labelling of NMNAT2 (red) and βIII-tubulin (green) in nerve fibers at various concentrations of MP. (**d**) NMNAT2-immunoreactive (ir) nerve fiber length normalised to βIII-tubulin-ir nerve fiber length in identical areas at various concentrations of MP. ** *p* < 0.005 vs. absence of MP (Williams’ test). Each value represents the mean ± standard deviation of more than three wells. MP: methylparaben; NMNAT2: nicotinamide mononucleotide adenylyltransferase 2.

**Figure 8:**
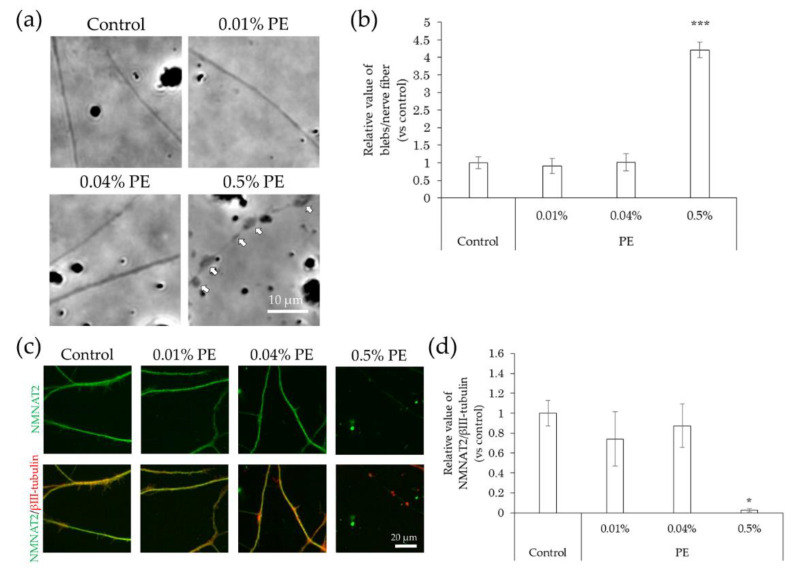
Effects of PE treatment on bleb formation and NMNAT2 expression in nerve fibers. After culturing for 3 days, the effects of the direct interaction between hiPSC-SN and PE were assessed. (**a**) Morphological changes in nerve fibers 24 h after treatment with PE. The arrows indicate blebs. (**b**) Number of blebs at various concentrations of PE. *** *p* < 0.001, vs. absence of PE (Dunnett’s test). (**c**) Double labelling of NMNAT2 (red) and βIII-tubulin (green) in nerve fibers at various concentrations of PE. (**d**) NMNAT2-immunoreactive (ir) nerve fiber length normalised to βIII-tubulin-ir nerve fiber length in identical areas at various concentrations of PE. * *p* < 0.05 vs. absence of PE (Steel’s test). All values represent the mean ± standard deviation of more than three wells. NMNAT2: nicotinamide mononucleotide adenylyltransferase 2; PE: phenoxyethanol.

In comparison with the control, the incidence of blebs in nerve fibers did not change upon exposure to methylparaben at concentrations of 0.0004%, 0.002%, and 0.01%, but increased significantly at a concentration of 0.05% (Figure 7a,b). NMNAT2 expression levels in nerve fibers tended to decrease slightly, but not significantly, at methylparaben concentrations of 0.0004% and 0.002% and decreased significantly at concentrations of 0.01% and 0.05% (Figure 7c,d). There was no change in the incidence of blebs in nerve fibers upon exposure to 0.01% or 0.04% phenoxyethanol, but there was a significant increase of 0.5% (Figure 8a,b). Upon exposure to phenoxyethanol, there was a slight but not significant decrease in blebs and a significant decrease in NMNAT2 expression in nerve fibers at concentrations of 0.01% and 0.04%, and at a concentration of 0.5%, respectively (Figure 8c,d).

**Figure 9:**
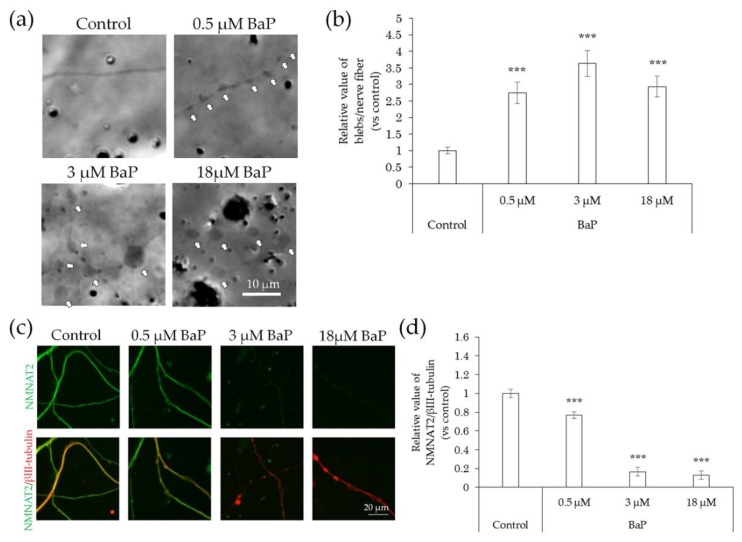
Effects of BaP treatment on bleb formation and NMNAT2 expression in nerve fibers. After culturing for 3 days, the effects of the direct interaction between hiPSC-SN and BaP were assessed. (**a**) Morphological changes in nerve fibers 24 h after treatment with BaP. The arrows indicate blebs. (**b**) Number of blebs at various concentrations of BaP. (**c**) Double labelling of NMNAT2 (red) and βIII-tubulin (green) in nerve fibers at various concentrations of BaP. (**d**) NMNAT2-immunoreactive (ir) nerve fiber length normalised to βIII-tubulin-ir nerve fiber length in identical areas at various concentrations of BaP. *** *p* < 0.001, vs. absence of BaP (Dunnett’s test). Each value represents the mean ± standard deviation of the three wells. BaP: benzo[a]pyrene; NMNAT2: nicotinamide mononucleotide adenylyltransferase 2.

In comparison with that of the control, the incidence of blebs increased following exposure to BaP at concentrations of 0.5–18 μM (Figure 9a,b). In contrast, NMNAT2 expression levels in nerve fibers decreased in a concentration-dependent manner (Figure 9c,d).

## 3. Discussion

In the present study, we found that methylparaben and phenoxyethanol promoted nerve fiber elongation. Preservatives and air pollutants are known to cross the epidermal barrier, which can accelerate aging [18] and cause inflammatory skin disorders [27,28,29]. When dispersed in the skin, these substances may directly interact with the nerve fibers. Parabens and phenoxyethanol have also been reported to be irritants capable of inducing nerve firing in electrophysiological experiments [30,31,32,33]. Our data suggest that methylparaben and phenoxyethanol may cause nerve elongation, even at the estimated concentration that has been reported to reach the epidermis with daily cosmetic use [18], which may be involved in the exacerbation of abnormal skin sensations such as hypersensitivity.

Furthermore, phenoxyethanol can activate transient receptor potential vanilloid 1 (TRPV1) [31], the activation of which promotes the nerve fibre elongation [31]. Thus, TRPV1 may play an important role in the elongation of nerve fibers upon exposure to phenoxyethanol. Methylparaben can also activate transient receptor potential ankyrin 1 (TRPA1) [32], but it has been reported to inhibit the elongation of nerve fibers in the central nervous system of rodents [33]. These findings suggest that different mechanisms exist in the peripheral and central nervous systems. Thus, methylparaben might affect the elongation of nerve fibers by activating receptors other than TRPA1 in the peripheral nervous system. Transient receptor potential (TRP) channels are involved in the development of skin sensations, such as pain and itch, and perception of temperature [34], suggesting that phenoxyethanol and methylparaben may excite sensory neurons via TRP channels and cause abnormal sensory transmission in the skin.

BaP is one of the components of air pollutants in diesel engine exhaust gas and cigarette smoke, capable of inducing oxidative stress in human skin [35] and possible inflammatory reaction [36]. Our results also indicate that BaP causes neurodegeneration without significant elongation of nerve fibers. It is known that damage to these fibers leads to increased spontaneous firing or changes in conduction or neurotransmitter properties [37]. Our results are consistent with previous reports showing decreased neurotransmission in rats treated with BaP [38] and a report of itching and numbness in patients who had ingested Yusho (a cooking oil contaminated with dioxins) [39]. Given that neurodegeneration without elongation of nerve fibers causes sensory abnormalities, evaluation of the safety of a substance should include not only its ability to cause hyperinnervation, but also its ability to cause neurodegeneration.

Itch hypersensitivity can lead to scratching and further deterioration of barrier function. Therefore, it is important to select cosmetic ingredients that do not exacerbate the hypersensitivity. In this study, we found that our in vitro model could be used to evaluate the morphological characteristics of sensory neurons, such as nerve elongation and/or degeneration, in the context of abnormal skin sensation. In fact, we were able to determine the direct effects of chemical substances on nerve fibers. Thus, our method may be used to detect chemicals that have the potential to exacerbate sensitive skin.

As methylparaben and phenoxyethanol are widely and safely used as preservatives worldwide, it is unlikely that cosmetics containing these ingredients will immediately cause the exacerbation of abnormal skin sensation with normal use. Moreover, long-term use of cosmetics containing these ingredients may cause them to accumulate and affect skin sensations. Further investigations of a wider range of cosmetic ingredients are required for a more precise understanding of their effects on nerve fibers in the epidermis and to provide useful information for developing safer cosmetics.

Recently, using mainly rat PC12 cell line, it was revealed that some natural ingredients, such as ginsenosides, curcumin, withanosides, and green tea polyphenols, promote nerve fiber elongation [40]. These natural compounds may be useful for neuronal regeneration in various forms of neuronal injury. However, little is known about natural ingredients that affect neuronal morphology such as nerve growth or nerve degeneration. For future study, using the hiPSC-SNs model, we aim to focus on the investigation of natural ingredients or their extracts for their effect on the inhibition of nerve elongation or nerve degeneration, which may help improve abnormal skin sensations.

## 4. Materials and Methods

### 4.1. Culture of hiPSC-SNs

The hiPSC-SNs (ReproCELL Inc., Tokyo, Japan) were cultured in ReproNeuro MQ medium (RCDN102; ReproCELL Inc., Tokyo, Japan) with 10 ng/mL nerve growth factor (NGF; 141-07601, FUJIFILM Wako Pure Chemical Corporation, Osaka, Japan) at 37 °C under 5% CO_2_.

For immunocytochemical characterization, the cells were seeded at a density of 4.0 × 10^5^ cells/cm^2^ in 24-well plates (MS-80240; Sumitomo Bakelite Co., Ltd., Tokyo, Japan) and incubated for 6 days at 37 °C under 5% CO_2_.

To assess the effect of NGF and chemical substances, cells were seeded at a density of 2.0–4.0 × 10^5^ cells/cm^2^ in 96-well plates (655090; Greiner Bio-One International GmbH, Kremsmünster, Austria). After 2 days of culture, the medium was replaced with ReproNeuro MQ medium containing one of the following: 0.0004% (0.03 mM), 0.002% (0.13 mM), 0.01% (0.66 mM), or 0.05% (3.3 mM) methylparaben (132-02635; FUJIFILM Wako, Osaka, Japan); 0.01% (0.7 mM), 0.04% (3 mM), or 0.5% (36 mM) phenoxyethanol (169-12072; FUJIFILM Wako, Osaka, Japan); or 0.5, 3, or 18 μM benzo[a]pyrene (BaP, 50-32-8; FUJIFILM Wako, Osaka, Japan) with 0.05% dimethyl sulphoxide (DMSO, 94563; Merck KGaA, Darmstadt, Germany); or 0.05% DMSO (as a control) and incubated for 24 h at 37 °C under 5% CO_2_.

To assess the effect of hydrogen peroxide (H_2_O_2_) after 2 days of culture at 37 °C under 5% CO_2_, the medium was replaced with ReproNeuro MQ medium containing 50 μM H_2_O_2_ and incubated for 2 h at 37 °C under 5% CO_2_, after which the spent medium was replaced with fresh medium (supplemented with NGF [10 ng/mL]) and incubated for 24 h at 37 °C under 5% CO_2_.

### 4.2. Immunocy to Chemistry

The hiPSC-SNs were fixed with 4% paraformaldehyde in a phosphate-buffered saline (PBS; pH 7.4) for 15 min at room temperature, permeabilised with 0.2% Triton X in PBS for 30 min, and then incubated with 1% bovine serum albumin in PBS for 1 h. This was followed by overnight incubation at 4 °C with primary antibodies against Brn3a (BAM1585; Millipore, Burlington, MA, USA), peripherin (AB1530; Millipore, Burlington, MA, USA), βIII-tubulin (801201; BioLegend, San Diego, CA, USA; 5666S; Cell Signalling Technology, Danvers, MA, USA), or nicotinamide mononucleotide adenylyltransferase 2 (NMNAT2; ab56980; Abcam, Cambridge, UK). The cultured neurons were incubated with secondary antibody solutions (Alexa Fluor 647 donkey anti-mouse IgG or Alexa Fluor 488 goat anti-rabbit IgG) and DAPI at dilutions of 1:500 and 1:5000, respectively, for 1 h at 4 °C. Immunoreactivity was observed using an automated image acquisition and analysis system (IN Cell Analyzer 2200; GE Healthcare Life Sciences, Marlborough, MA, USA). The immunoreactive (ir) nerve fiber length was quantified using an image analyser IN Cell investigator 1.6 (GE Healthcare Life Sciences, Marlborough, MA, USA), which can automatically trace and measure nerve fiber length without measuring cell bodies or debris.

### 4.3. Quantitative Evaluation of Neuronal Differentiation

More than five immunocytochemical images (0.4 × 0.4 mm) in each experiment were captured at the indicated times. The number of neurons positive for DAPI, βIII-tubulin, peripherin, and Brn3a in each image was counted manually.

### 4.4. Quantitative Evaluation of Nerve Degeneration

More than three phase-contrast images in each well were captured at the indicated times. The numbers of blebs and nerve fibers in nine 0.1 × 0.1 mm boxes from each image were counted manually. Only blebs more than twice the width of the associated nerve fibers were counted. The ratio of blebs to nerve fibers in each box was then calculated according to a previously described method [23].

### 4.5. Quantitative Analyses of Nerve Fiber Length

The hiPSC-SNs were stained with 250 nM NeuO (ST-01801; STEMCELL Technologies, Vancouver, BC, Canada), a neuron-selective probe [17], and 0.7 mM Hoechst 33, 342 (PG023; Dojindo Molecular Technologies, Inc., Kumamoto, Japan). After incubation for 1 h, more than 36 fluorescent images (0.7 × 0.7 mm) in each well were obtained using IN Cell Analyzer 2200. The nerve fiber length was measured using an image analyser (IN Cell investigator 1.6). To calculate the average nerve fiber length per cell, an image analyser was used to automatically count the number of cells in an identical area.

### 4.6. Statistical Analyses

Statistical analyses were performed using EZR (Saitama Medical Centre, Jichi Medical University, Tokyo, Japan), which is a graphical user interface for R (The R Foundation for Statistical Computing, version 2.13.0, Vienna, Austria). First, the Bartlett’s test confirmed the homoscedasticity of the population of each group. Next, the Dunnett’s test was used when homoscedasticity was assumed, and the Steel’s test was used when it was not assumed to compare with the control group. For multiple comparisons between all groups, the Tukey’s test was used after confirming that homoscedasticity was assumed. For gradient multiple comparisons, Williams’ test was used after confirming that homoscedasticity was assumed. In all analyses, the statistical significance was set at *p* < 0.05.

## 5. Conclusions

In conclusion, our findings suggest that methylparaben and phenoxyethanol promote nerve elongation in hiPSC-SNs, whereas benzo[a]pyrene induces nerve degeneration. These alterations may be at least partly involved in the onset and progression of abnormal skin sensation in humans. Thus, hiPSC-SNs may be a useful model for investigating the effects of exposure to different substances and microenvironments on nerve fibers and for evaluating the safety of ingredients in cosmetics and improving the quality of life in people with abnormal skin sensation.

## Data Availability

The datasets generated or analysed during the current study are available from the corresponding author upon reasonable request.
